# Review article: the human intestinal virome in health and disease

**DOI:** 10.1111/apt.14280

**Published:** 2017-09-04

**Authors:** S. R. Carding, N. Davis, L. Hoyles

**Affiliations:** ^1^ Norwich Medical School University of East Anglia Norwich UK; ^2^ The Gut Health and Food Safety Research Programme The Quadram Institute Norwich Research Park Norwich UK; ^3^ Department of Surgery and Cancer Imperial College London London UK

## Abstract

**Background:**

The human virome consists of animal‐cell viruses causing transient infections, bacteriophage (phage) predators of bacteria and archaea, endogenous retroviruses and viruses causing persistent and latent infections. High‐throughput, inexpensive, sensitive sequencing methods and metagenomics now make it possible to study the contribution dsDNA, ssDNA and RNA virus‐like particles make to the human virome, and in particular the intestinal virome.

**Aim:**

To review and evaluate the pioneering studies that have attempted to characterise the human virome and generated an increased interest in understanding how the intestinal virome might contribute to maintaining health, and the pathogenesis of chronic diseases.

**Methods:**

Relevant virome‐related articles were selected for review following extensive language‐ and date‐unrestricted, electronic searches of the literature.

**Results:**

The human intestinal virome is personalised and stable, and dominated by phages. It develops soon after birth in parallel with prokaryotic communities of the microbiota, becoming established during the first few years of life. By infecting specific populations of bacteria, phages can alter microbiota structure by killing host cells or altering their phenotype, enabling phages to contribute to maintaining intestinal homeostasis or microbial imbalance (dysbiosis), and the development of chronic infectious and autoimmune diseases including HIV infection and Crohn's disease, respectively.

**Conclusions:**

Our understanding of the intestinal virome is fragmented and requires standardised methods for virus isolation and sequencing to provide a more complete picture of the virome, which is key to explaining the basis of virome‐disease associations, and how enteric viruses can contribute to disease aetiologies and be rationalised as targets for interventions.

## 1 THE HUMAN VIROME

Viruses are thought to be the most abundant and diverse entities on Earth, numbering as many as 10^31^ virus‐like particles (VLPs),^1^ though this paradigm is likely to change in light of information from large‐scale sequencing surveys of marine environments and improved analytical tools.^2^ With the advent of new, sequence‐based technologies that do not rely on the ability to isolate viruses for their identification, it is now possible to define and characterise viruses in different environmental samples in greater detail than ever before, which has resulted in an increased interest in the role the viral assemblage of the human gut microbiota plays in health and disease. The following reviews our current knowledge on the human intestinal virome.

**Figure 1:**
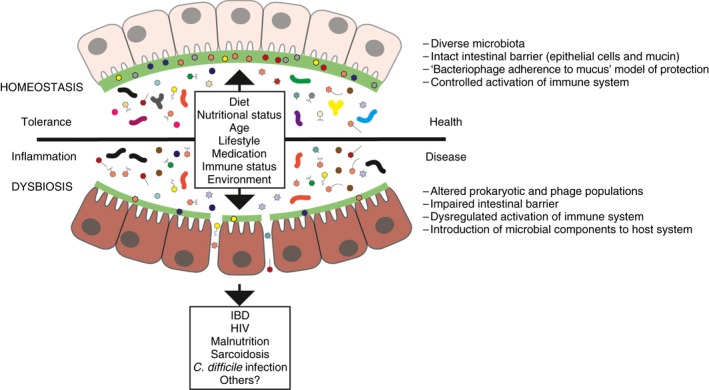
The mammalian intestinal virome comprises viruses that infect eukaryotic and prokaryotic cells. It is established soon after birth and is dominated by viruses that infect bacteria (ie, phages). The virome establishes a mutualistic relationship with eukaryotes/prokaryotes, contributing to intestinal homeostasis by influencing microbial ecology and host immunity. Composition of the virome is influenced by numerous factors that affect viruses directly (infection) or change host‐cell populations (eg, antibiotics, diet). Members of the virome may contribute to the pathogenesis of certain diseases via microbial host lysis leading to dysbiosis, infection of epithelial cells, and/or translocation of the compromised or damaged mucosal barrier to gain access to underlying tissues and immune cells, leading to immune activation. Dysbiosis can be defined as a microbial imbalance or any changes to the composition of resident microbial communities relative to the community found in healthy individuals.^101^, ^102^, ^103^ Virome association with certain disease states is characterised by changes in diversity, and predominance of specific virotypes (eg, members of the order *Caudovirales* in IBD)

A virome comprises all the nucleic acids (ssDNA, dsDNA, ssRNA and dsRNA) belonging to the VLPs associated with a particular ecosystem. The human virome is a genetically complex component of the microbiome, with the blood, nose, skin, conjunctiva, mouth, vagina, lungs and gastrointestinal (GI) tract harbouring their own distinct virus assemblages (Table S1). The genetic content of VLPs comprising bacteriophages (phages) that infect bacteria and archaea and, to a much lesser extent, human‐, plant‐, amoebae‐ and animal‐infecting viruses found along the GI tract constitute the human intestinal virome (Figure 1).

## 2 BACTERIOPHAGES

Phages can be lytic or lysogenic. Lytic (virulent) phages bind to specific host‐cell receptors, then penetrate and infect their host cell to hijack its replication and translation machinery to produce virions; once sufficient virions have accumulated (tens to thousands, depending on the phage), the lytic enzymes they produce cause the host cell to lyse, releasing the virions into the surrounding environment where they can infect new host cells. Lytic phages can have narrow or broad host ranges, infecting only one strain of a prokaryote or multiple species of closely related prokaryotes. These entities have been used in the past to treat infections, and have recently been revisited as alternatives to antibiotic therapies for a range of infections and as means of controlling food‐contaminating bacteria such as *Listeria* spp.^3^ Their gene products are also of interest in biotechnological and medical applications.^4^ Lysogenic (temperate) phages do not kill their host but instead integrate into their host's genome without interfering with its replication, incorporating the phage into its genome as a prophage that is transmitted to its progeny at each cell division. Lysogenic phages can be converted to a lytic cycle in response to environmental stressors (eg, antibiotics).

Phage‐host interactions influence host and viral evolution. The ability of phages to transfer genes from one prokaryotic host to another can lead to increased diversification of viral species, and increased antibiotic resistance and/or induction of toxins or virulence factors in prokaryotes.^5^ Some phages alter the antigenicity of their hosts by producing enzymes that modify the O‐antigen component of lipopolysaccharides.^6^ Modification of surface structures of prokaryotes has the potential to affect microbial interactions with the human host, and influence niche specialisation within the GI tract.^7^ Presence of clustered regularly interspaced short palindromic repeats (CRISPRs) confers upon prokaryotes resistance to phage infection and contributes to prokaryotic adaptive immunity. Analyses of metagenomic sequence data provide detailed information on phage‐host and phage‐phage competition within the human faecal microbiome, implying CRISPR spacers are actively and continuously acquired by prokaryotes in response to the presence of phages in the GI tract.^8^ The potential effects of such phage‐host interactions on microbiota composition/function or host health are unknown.

## 3 CHARACTERISING THE HUMAN INTESTINAL VIROME

Viruses do not encode universally conserved genes such as the 16S or 18S rRNA genes of prokaryotes and eukaryotes, respectively, and are genetically highly diverse. Consequently, it is not possible to use metataxonomic approaches such as 16S rRNA gene sequencing to characterise VLPs within ecosystems. Traditionally, classical approaches—mainly microscopy and cultivation—have been relied upon to characterise VLPs in the human gut.

**Figure 2:**
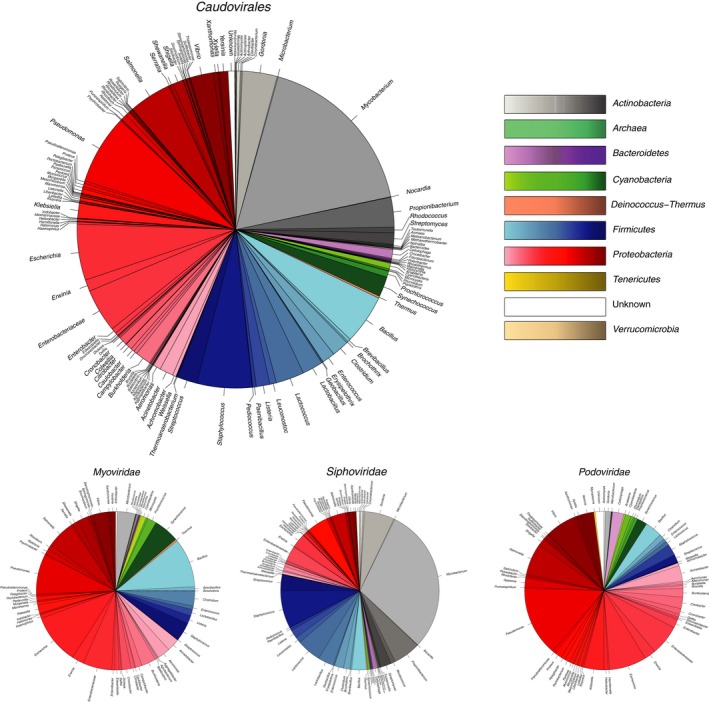
Breakdown of complete genomes of members of the order *Caudovirales* (dsDNA viruses, no RNA stage) available from NCBI Genome on November 21, 2016 (n = 1943). The number of publicly available *Caudovirales* genomes had only increased to 2044 by July 6, 2017. Genera of prokaryotes (with some families and higher taxa) infected by phages are shown around the chart; colours correspond to phyla (kingdom in the case of the *Archaea*) of prokaryotes infected by different phages. Aggregated data are shown for each of the three families of the order *Caudovirales* in the large plot. *Myoviridae*, n = 538 complete genomes; *Siphoviridae*, n = 1063 complete genomes; *Podoviridae*, n = 342 complete genomes. Larger versions of the pie charts shown in this figure are available in Supporting Information

Based on transmission electron microscopy (TEM), mucosal samples contain ~1.2 × 10^9^ VLPs/biopsy.^9^ VLPs have been detected in caecal contents at 10^6^/mL using TEM^10^ with faeces harbouring 10^8^‐10^9^ VLPs/g wet weight.^10^, ^11^ In all GI contents examined to date by microscopy, the overwhelming majority of VLPs have been phages of the order *Caudovirales*, with the human GI tract estimated to harbour 10^15^ phages in total.^3^, ^9^, ^10^, ^12^ The order *Caudovirales* encompasses most known phages (Figure 2), comprising the families *Myoviridae* (nonenveloped, head‐tail structure, contractile, dsDNA genomes of 30‐250 kb), *Siphoviridae* (nonenveloped, head‐tail structure, noncontractile, dsDNA genomes of ~50 kb) and *Podoviridae* (nonenveloped, head‐short tail structure, dsDNA genomes of 40‐42 kb). Every individual harbours a morphologically unique phage population.^10^, ^12^

For many years, the isolation of gut‐associated phages was confined to samples derived from sewage or water sources, with the presence of phages considered indicative of nonspecific (animal, human) faecal contamination. Recently attention has turned to isolating phages directly from human microbiota samples (Table 1). While in its infancy, this work holds great promise for improving our knowledge of the gut virome, will facilitate metagenomic studies and aid in vitro and in vivo studies investigating the influence of phages on prokaryote populations in the human gut. Significant improvements to isolation methods are needed to allow the efficient and reliable isolation and characterisation of phages infecting fastidious anaerobic prokaryotes. In addition, no single isolation approach is applicable to all prokaryotes, and based on evidence from *Bacteroides* spp. it is likely gut‐associated phages have very narrow host ranges.^7^

**Table 1:** Phages recently isolated from the human gut microbiota

Host bacterium	Source of host bacterium	Phage(s); source	Lytic/lysogenic
*Klebsiella pneumoniae* L4‐FAA5^82^	Caecal effluent	KLPN1^a^; caecal effluent	Lytic
*Clostridium difficile* HMC114^83^	Faeces	Phage tail‐like particle; *Clostridium difficile* HMC114	Lysogenic
*Escherichia coli* DPC6051^84^	Unknown	ɸAPCEc01, ɸAPCEc02, ɸAPCEc03; faeces	Lytic
*Proteus mirabilis* CEMTC 73^85^	Faeces of a patient after bariatric surgery	PM16^a^; faeces	Lytic

a Phage isolated from same sample as host strain.

Study of viruses in clinical settings will be facilitated by methods such as the Virome Capture Sequencing platform for Vertebrate viruses (VirCapSeq‐VERT), a system that uses ~2 million probes targeting the genomes of 207 viruses known to infect humans and other vertebrates to capture viral nucleic acids present in blood and tissue homogenates.^13^

## 4 METAGENOMICS AND THE HUMAN INTESTINAL VIROME

The only reliable molecular method currently available for routine surveys of the human virome is metagenomics. Metagenomics is a culture‐independent, molecular‐based approach that allows functional and sequence‐based analyses of the collective microbial genomes contained in an environmental sample, providing a powerful approach for exploring the ecology of complex microbial communities.^14^ It has been used to examine prokaryotic communities associated with the human gut microbiome in health and disease,^15^, ^16^, ^17^, ^18^ and to examine viromes associated with different regions of the human body (Table S1).

A protocol involving homogenisation of faeces in buffer, centrifugation (to remove cell debris), tangential flow filtration (TFF) (to concentrate large‐volume samples and isolate VLPs), ultracentrifugation and metagenomic reconstruction was used to characterise the first human faecal virome.^19^ However, recent improvements in recovery methods give the potential to characterise human‐associated VLP assemblages at the molecular level in greater detail than ever before (Table 2). These methods have yet to be compared with one another directly, and the issue of which DNA extraction method or kit is best for use with gut virome samples has not been addressed. To avoid the problems that have beset studies of the prokaryotic component of the gut microbiome with respect to reproducibility, contaminants and comparable approaches across studies and datasets, it is important the virome community adopts robust, standardised methods at the earliest opportunity.

**Table 2:** Methods used for recovering VLPs from intestinal/faecal contents

Sample(s)	Comments
Caecal and faecal^10^	Filtration and polyethylene glycol (PEG) precipitation to concentrate VLPs, with and without CsCl centrifugation 0.45 μm filters instead of 0.22 μm doubled the number of VLPs and the quantity of DNA recovered from samples Used pulsed‐field gel electrophoresis to demonstrate differences in viromes among individuals
Faecal^86^	ViSeq (VIDISCA enrichment of VLPs/conversion of RNA to cDNA/SLIM) Documented HIV‐1 and overall viral content of faecal samples of late‐stage AIDS patients
Faecal^12^	Microfiltration‐free PEG and TFF methods optimised Spiked samples with low numbers of c2 (*Siphoviridae*), ϕ29 (*Podoviridae*), and T4 (*Myoviridae*) phages to determine phage recovery PEG more effective than TFF, yielding 16 times more phage particles and 68 times more phage DNA per volume Increased recovery of *Caudovirales* at the expense of eukaryotic viruses
Mock virome containing 9 phages/viruses^66^	NetoVIR (novel enrichment technique of viromes) Homogenisation with glass beads led to destruction of viruses Increasing centrifugation times at 17,000 ***g*** led to reductions in virus recovery, possibly due to presence of aggregates in the sample Filtering through 0.2, 0.45 or 0.8 μm filters led to significant losses of mimivirus; 0.2 μm filter led to significant loss of Herpesvirus Chloroform treatment led to losses of enveloped viruses (coronavirus and mimivirus) and nonenveloped viruses (rotavirus and polyomavirus) Demonstrated effect of increasing number of PCR cycles on recovery of viruses in the mock community (ie, lower yields of some viruses as number of cycles increased) No data presented on NetoVir when used on gut/faecal samples
Faecal^87^	Filtered (0.2 μm) sample subjected to fluorescence‐activated cell sorting (FACS) Only looked at a small fraction of the faecal virome, but different viral fractions could theoretically be separated using FACS Allowed direct sequencing of viral DNA without the need for whole‐genome amplification Avoided contamination with bacteria Led to improved assembly of viral genomes
Artificial community of 25 viruses^88^	Mock community contained dsDNA (*Adenoviridae*,* Herpesviridae*), dsRNA (*Reoviridae*), and ssRNA (*Astroviridae*,* Caliciviridae*,* Coronaviridae*,* Picornaviridae*,* Orthomyxoviridae*,* Paramyxoviridae*) viruses Filtration and nuclease digestion had little effects on overall results Pre‐amplification of nucleic acids prior to sequencing led to more biased genome coverage with near‐duplicate reads accumulating over the same region, decreasing genome coverage ScriptSeq > TruSeq > Nextera for virus detection and genome coverage Maximum of 22/25 viruses recovered in sequenced viromes
Artificial intestinal microbiota (six phages, two bacteria^67^	CsCl centrifugation most efficient for removing host DNA, but discriminated against some phages and was not reproducible; not suitable for quantitative studies Did not process PEG‐precipitated samples for sequencing Recommended using filtration + DNase, or dithiothreitol treatment + filtration + DNase to collect VLPs, but did not test on human faeces Cautioned about over‐interpreting data: abundances of individual phages within samples deviated by more than one order of magnitude from the actual input number of phage particles

The first faecal metagenome was generated from the faeces of a 33‐year‐old healthy male using a clone‐based strategy.^19^ It was predicted to comprise ~1,200 virotypes, with the most abundant virus making up 4% of the total. Siphophage sequences made up the majority of recognisable viruses, with 59% of the sequences not related to anything within public databases. Sequences with significant matches to myophages, podophages and microphages were also detected. The most common sequence matches were with phages associated with *Listeria monocytogenes*,* Burkholderia thailandensis* and *Lactococcus lactis*. Prophages within bacterial genomes also made a large contribution to the recognisable viruses identified.^19^ Numerous sequences with similarity to phages that infect Gram‐positive bacteria were also detected, reflecting results from studies on faecal bacterial populations that have shown *Firmicutes* and, to a lesser extent, *Actinobacteria* are among the most predominant bacteria in human faeces. This suggested the most abundant phage groups in a given environment reflect the kinds of microbes found in that environment.^1^

## 5 MINING TOTAL COMMUNITY METAGENOMES TO REVEAL VIRUS DIVERSITY

With the exceptions of the *Proteobacteria*, mycobacteria and some lactic acid bacteria, very few phages infecting prokaryotes have been isolated and sequenced (Figure 2). Between 4% and 22% of DNA in total community metagenomes can be derived from VLPs.^20^, ^21^, ^22^ This allows potential recovery of phage sequences not well represented in virome studies, though still excludes RNA viruses from detailed analyses.

Human gut‐specific *Bacteroides* phage φB124‐14 was isolated from municipal waters and infects *Bacteroides fragilis* GB‐124 and a limited range of other *B. fragilis* strains.^7^, ^23^ Sequences with homology to this phage were found in faecal metagenomes from MetaHIT (104/124) and Japan (3/13), and human gut viromes (2/16). Sequences associated with this phage were not found in American gut‐associated datasets (metagenome or virome), suggesting geographical variation in distribution of gut‐associated phages.^7^ Using a similar metagenomic mining approach but with major capsid protein VP1 specific for the ssDNA phage family *Microviridae*, members of the subfamilies *Gokushovirinae*,* Alpnavirinae* and *Pichovirinae* were detected in faecal metagenomes.^24^

By using phage genome signature‐based recovery with metagenomes, several (predominantly temperate) potential gut‐specific phages infecting members of the order *Bacteroidales* were identified.^21^ This alignment‐free approach was able to resolve phage sequences not readily detected by conventional alignment‐driven approaches. The main limitation of this method is the lack of available genome sequences from isolated phages, as “driver sequences” are required to provide baseline information upon which inferences can be made regarding sequence data and host phylogeny (only four such “driver sequences” were required in the *Bacteroidales* study).^21^ Using co‐occurrence profiling, crAssphage was identified in publicly available metagenomes, and by CRISPR analysis and co‐occurrence profiling predicted to infect *Bacteroides* or *Prevotella* spp.^22^ This phage, with a 97,065 bp genome encoding 80 proteins, is predicted to make up to 24%‐90% of all reads in faecal viromes, and 1.7% of reads in all human faecal metagenomes (up to 22% in the HMP dataset). Long‐range PCR and Sanger sequencing were used to confirm the presence of the genome in a faecal sample. The role of this phage in the human gut is unknown, and a representative of it remains to be isolated and purified.

Novel human‐associated viruses present in the gut virome can also be detected using viral metagenomics. Analyses of diarrhoea samples from Dutch patients with unexplained acute gastroenteritis led to the identification of novel members of the families *Anelloviridae*,* Picobirnaviridae*,* Herpesviridae* and *Picornaviridae*.^25^ The role of these viruses in gastroenteritis is still to be established. It is also important to remember in exploratory (and other) studies that detecting virus nucleic acids in samples gives no indication of whether the viruses infect human or other cells.^26^

## 6 INDIVIDUAL INTESTINAL VIROMES ARE UNIQUE, BUT SOME PHAGES ARE SHARED AMONG INDIVIDUALS

A longitudinal study (three time points over 1 year) of four pairs of adult monozygotic twins and their mothers demonstrated that, while the faecal prokaryote communities of related individuals are very similar, each individual harbours a unique faecal virome.^27^ Intrapersonal diversity of the virome was low, with >95% of viruses retained over a 1‐year period, with a few temperate phages dominating the viromes. This contrasts with Lotka‐Volterra predator‐prey relationships, in which cyclic changes in phage and prokaryote abundance would be expected, or with kill‐the‐winner dynamics, in which detectable changes in phage‐host pairs would be detected. The majority (81%) of sequenced reads did not match any known viruses, and only 3.2 ± 2.8% reads could be assigned functions using KEGG. The predicted hosts of the identifiable phages were members of the phyla *Bacteroidetes* and *Firmicutes*, in accordance with the predominant bacteria detected in the samples by 16S rRNA gene sequencing. The ratio of virotypes to bacterial phylotypes was predicted to be ~1:1.

Assembly of sequence data from four deep‐sequenced DNA viromes (8.1 Gb sequence data in total) from two healthy individuals led to recovery of 4301 contigs, representing 72 complete phage genomes plus potentially complete and partial phage genomes.^28^ A minority (1703) of the contigs and 63/72 complete genomes contained at least one ORF with homology to a phage protein. Only 314 of all contigs contained homologues that allowed their assignment to a higher phage taxon, with order *Caudovirales* predominating. Using a low‐stringency approach (ie, recruitment of a single sequence read to any of the 4301 contigs) to detect genomes in samples, 17% of the contigs were found to be present in both individuals in at least one sample and 7% were present in all four samples. Sequence reads from ~20% of 62 faecal viromes (generated from healthy cohorts in three American cities and Cambridge, UK^29^) mapped to 160 contigs. A consortium of 155 phages contributed to the “healthy gut phageome” (HGP), though only 23 phages were present in >50% of samples (“core” phages, including crAssphage and nine other complete genomes), and 132 were present in 20%‐50% of samples (“common” phages); 1679 were present in 2%‐19% (“low overlap” phages). The HGP represented only 4% of the total phage community, with the expectation that the HGP will increase in size by analysing additional healthy individuals using deep sequencing.^28^ Although it has been proposed the HGP may play a role in maintaining and possibly restoring a dysbiotic microbiota,^30^ how this might be achieved, and the role and mechanisms by which shared communities of phages contribute to gut health, remains a matter of debate.

## 7 INTESTINAL VIROME VARIATION AND STABILITY

Interindividual variation among and intra‐personal stability of faecal viromes was confirmed in six healthy individuals enrolled in a dietary intervention study (two high‐fat/low‐fibre diet; three low‐fat/high‐fibre diet, one ad‐lib diet).^20^ Greater sequencing depth was achieved compared with earlier studies (336 Mb vs 70 Mb across all samples,^19^, ^27^ with 98% of sequence reads having no known hits in databases. Greater sequencing depth allowed assembly of contigs (7175 of at least 500 bp in length), with 87% of reads contributing to these. PHACCS (PHAge Communities from Contig Spectrum)‐predicted median species richness of the samples was 44 (range 19‐785), in agreement with Reyes et al^27^ (median of 35, range 10‐984). Tailed phages predominated (*Siphoviridae*, 18% of the total; *Myoviridae*, 10%; *Podoviridae*, 4.8%; *Microviridae*, 0.9%; other families, 0.4%). The majority of contigs (55%) shared no significant amino acid homology with any phage family, while 11% had homology to multiple phage families. No eukaryotic viruses were detected. Minor convergence of viromes was seen when individuals were fed the same diet, though each individual retained a unique virome. Induction of lysogeny may have contributed to changes in phage abundance during the dietary intervention, though this has not been demonstrated empirically. In contrast to total faecal metagenomes, which were enriched in genes for synthesis (amino acid, carbohydrate precursors), translational machinery and cell component biogenesis, VLPs were enriched for genes associated with replication, recombination, repair and unknown functions; consistent with viruses recruiting host‐cell machinery for their propagation. Findings regarding prophages were not in accord with previous studies (they did far more in‐depth analyses than previous studies): 2814 contigs had significant amino acid homology with an ACLAME prophage sequence, 41% were assigned to *Firmicutes*, 0.9% were assigned to *Bacteroidetes*, 16% were assigned to the *Proteobacteria*.^20^ This pattern did not match abundance of bacteria within samples, leading to the suggestion prevalence of temperate phages in faeces may differ among different phyla with the caveat that conclusions were dependent on phage sequences available in public databases at the time of the study. Although it is acknowledged overall individual faecal viromes are dissimilar, analyses of combined virome^20^, ^27^ and total faecal metagenome data suggest the existence of a nontrivial common reservoir of phages shared among individuals in different geographical regions.^8^

Because of the small size of virus genomes compared with prokaryote genomes, it is relatively easy to achieve greater sequencing depth than total metagenome studies. Deep sequencing of faecal viromes (48 Gb of virome DNA across 12 individuals at three different time points over a 2‐month period; ~1.3 Gb of sequence per virome) allowed empirical assessment of hypervariation driven by a unique reverse‐transcriptase (RT)‐based mechanism in faecal viromes.^31^ Between 573 and 3390 contigs longer than 1 kb were assembled from the donors' viromes. The majority of predicted genes (72%) within these contigs encoded unknown proteins, but as with previous studies the majority of sequences were associated with DNA phages. Only one donor harboured a eukaryotic DNA virus—*Human papilloma virus* type 6b. This is typical of faecal viromes of healthy humans, in which recovery of DNA eukaryotic viruses is rare.^19^, ^20^, ^27^, ^32^, ^33^ “Diversity‐generating retroelements” (DGRs), RT‐based systems that introduce mutations at adenines in specific repeated sequences,^33^ were identified in the viromes of 11/12 subjects studied. Six predicted hypervariable genes encoded Ig‐superfamily β‐sandwich domains that have been associated with viral evasion of the immune system^31^ and binding of phages to host mucin glycoproteins.^34^ Phage enrichment within the mucosa is thought to protect the host from bacterial infections by limiting the presence of mucosal bacteria (“bacteriophage adherence to mucus” model of protection), providing nonhost‐derived immunity operating throughout the GI tract.^34^

## 8 EVOLUTION OF THE INTESTINAL VIROME, AND THE INFLUENCE OF ANTIBIOTICS

In‐depth analysis of one healthy adult's faecal virome over 2.5 years (56 Gb of DNA sequenced over 24 samples taken at 16 time points) demonstrated its rapid evolution.^33^ The majority (80%) of 478 assembled contigs persisted over the duration of the study, and accumulated sequence variation over time. The stability of phage nucleotide sequences was family‐dependent. Temperate phages had the lowest substitution rate, whereas *Microviridae* had high substitution rates (>10^−5^ per nt per day), with identification of new viral species over the study period; numerous new viral species may emerge in the human gut over the course of a lifetime. Evolution of DGRs and CRISPRs in phage contigs was also observed. Therefore, multiple mechanisms contribute to phage sequence variation within the human faecal virome. Antibiotic therapy influences viromes, not by affecting VLPs directly but by affecting their prokaryote hosts.^35^ For example, quinolones have been shown to induce Shiga‐toxin‐encoding prophages by *Escherichia coli* in mice, leading to toxin production and death of animals.^36^ In a longitudinal study involving four individuals on various broad‐spectrum, long‐term intravenous antibiotic therapies for *Staphylococcus* infections, oral viromes were affected more than faecal viromes by antibiotic treatment, with an increase in representation of papillomaviruses in the oral virome of antibiotic‐treated patients.^35^ A nonsignificant increase in antibiotic‐resistance genes was seen in the faecal viromes of the patients. It was suggested sampling of individuals should be done at more regular intervals to get a true picture of the effect of antibiotics on viromes. It should also be noted the study was hampered by confounders relating to age of patients compared with controls and the patients being on different treatment regimes.

Willner et al^37^ first suggested the sharing of VLPs among individuals in their study of sputum samples from individuals with lung disease (Table S1). Later work on oral and faecal viromes of 20 individuals over a 6‐month period confirmed this, demonstrating phages were persistent in these viromes and readily shared among related and unrelated members of the same household.^38^ Sharing of phages encoding virulence or antibiotic‐resistance genes has implications for shaping of microbiomes of those in close contact with one another, and warrants further study. With respect to antibiotic‐resistance genes, their presence may have been over‐estimated in human viromes^11^, ^20^, ^27^, ^31^, ^35^, ^37^ as these entities are thought to be rarely encoded in phages.^39^ Consequently, care should be taken when analysing data: proper in silico checks with conservative cut‐offs are required to refine functional assignments and avoid over‐interpretation of data.^39^ Induction of prophages encoded in prokaryote genomes during antibiotic treatment appears to be more relevant to gut microbial ecology—with an increased level of generalised transduction and low level of transfer of antibiotic‐resistance genes.^39^

## 9 ssDNA VIRUSES IN THE INTESTINAL VIROME

To date, only one study has specifically examined the contribution of ssDNA viruses to the human faecal virome.^11^ *Microviridae* were found to represent the majority (27%‐49%) of known phage sequences in samples from five healthy South Koreans. This overestimation of *Microviridae* abundance was due to preferential amplification of small circular genomes by the phi29 polymerase used to amplify the ssDNA. Corrected abundances suggested the *Microviridae* represent 3%‐9% of known viruses in human faeces. Sequence analyses of a major capsid protein of the *Microviridae* and gut bacteria predicted *Prevotella* and *Bacteroides* to be the hosts of a number of these viruses, sharing high sequence similarity with prophage‐like sequences encoded by these bacteria. Newer protocols for amplification and sequencing of ssDNA from environmental samples will improve characterisation of this group of viruses within the human gut.^40^, ^41^, ^42^

## 10 RNA PHAGES IN FAECES OF HEALTHY ADULTS

The GI tract of mammals is one of the natural habitats of RNA phages, with special relationships appearing to exist between specific RNA phage groups and their hosts.^43^ Coliphage RNA viral assemblages in the faeces of cows, foxes, elephants, pigs, horses, birds and humans and found group II and III RNA phages were predominant in human faeces, while group I and II RNA phages were predominant in the faeces of other animals. Of the 597 human faecal samples examined, 76.5% had fewer than 10 pfu/mL sample and 14.6% had between 10 and 100 pfu/mL sample (20%, w/v, faecal homogenates).

Metagenomics was used to examine RNA viral assemblages in faecal samples obtained from two healthy adults: two samples were collected from one donor 6 months apart, with one sample obtained from the other donor.^44^ Three shotgun viral cDNA libraries were generated, with a total of 36,769 viral sequences obtained; 91.5% of these had significant similarity to sequences in the GenBank database, with three‐quarters being most similar to viruses. Most (25,040/25,779 known) sequences were similar to plant‐pathogenic RNA viruses. Forty‐two viral species (2 animal, 35 plant, 1 yeast and 4 phage) were identified in the faecal RNA viral libraries, with *Pepper mild mottle virus* (PMMV), a well‐characterised plant‐pathogenic virus that infects all species of the genus *Capsicum*, being the most abundant. Using RT‐PCR, PMMV was detected in food and faecal (10^6^‐10^9^ virions/g dry weight faeces) samples from three different individuals. Prevalence of PMMV in the human population was also demonstrated, being detected in faecal samples from North American and Singaporean (both 6/9 positive) individuals. In addition to PMMV, 24 of the 35 plant‐pathogenic viruses were associated with consumable products such as cereals, fruits, tobacco and vegetables, suggesting a large proportion of faecal RNA viruses come from exogenous sources. Support for this hypothesis came from the detection of PMMV in some processed pepper‐containing foods. The amount of virus detected in faeces exceeded that detected in the ingested foodstuffs, suggesting plant RNA viruses replicate in the human GI tract; however, no data were presented to support this hypothesis. Faeces‐derived PMMV could infect wax pepper plants, suggesting humans and other animals could act as transmission vectors for plant viruses through their digestive tracts. A second study^45^ confirmed the presence of PMMV, and other plant RNA viruses, in human faeces. Studies into the ability of plant viruses to replicate in the human gut (in epithelial and microbial cells) should be initiated.^44^, ^45^

The low numbers of phage sequences retrieved (corresponding to *Haemophilus influenza* phage HP2, Roseophage SIO1, phage L cro and *Shigella flexneri* phage V^44^ and enterobacteria phage phiK and phage phiV10^45^) reflected earlier findings^43^ in relation to the low numbers of RNA phages recovered from human faeces.

## 11 ESTABLISHMENT OF THE FAECAL VIROME

Microscopic examination of the meconium (ie, first faecal sample) of a newborn infant did not detect any VLPs.^32^ By the end of the first week of life, ~10^8^ VLPs/g wet weight faeces could be detected, with this number seen as an underestimation of the true number due to high background fluorescence and clumping of the sample. A viral metagenome was generated from the faeces of the 1‐week‐old infant. Similar to adult DNA viral metagenomes, siphophage and prophage sequences predominated. Eight viral genotypes were predicted to be present in the infant's faecal virome, with the most abundant viral genotype contributing 43.6% to the total. The low diversity of the infant faecal virome correlated well with the known low diversity of the faecal bacterial assemblage in the first week of life. Sequences with significant similarity to podophages and myophages were also detected. More than 50% of the significant hits were to phages that infect Gram‐positive bacteria (*Lactobacillus*,* Enterococcus*,* Bacillus*,* Lactococcus*,* Streptococcus* and *Staphylococcus*), with hits to phages that infect Gram‐negative bacteria (*Bacteroides* and *Listonella*) also identified. Microarray analyses of viromes obtained from faecal samples at weeks 1 and 2 of life with probes constructed from the week 1 sequencing library showed the temporal nature of the infant gut virome, with >50% sequences that were at high abundance at week 1 absent at week 2, and the most abundant sequences at week 2 being of medium abundance at week 1. No sequences were present at high levels at week 1 or 2, demonstrating turnover of the most abundant viral sequences over a short period of time.

Primers designed based on the sequences of the three largest contigs generated from sequencing library amplified sequences corresponding to a *Streptococcus pyogenes* phage terminase (large subunit), a *Bacteroides thetaiotaomicron* integrase and *Bacillus thuringiensis* phage MZTP02. A portion of the sequence amplified by the *B. thetaiotaomicron* integrase primer set was identical to a viral sequence retrieved in the first adult faecal virome study.^32^ VLP DNA was isolated from the breast and formula milk fed to the infant, faecal samples obtained at 1, 2, 5 and 14 weeks of age, and from the faeces (two samples taken a year apart) of an unrelated adult. All three VLP sequences were present in the first 3 months of the infant's life and did not originate from breast or formula milk. The *B. thetaiotaomicron* integrase was detected in all faecal (infant and adult) samples examined, while the other two VLP sequences were detected only in the infant faecal samples. It is possible the assay used in this study was not sensitive enough to detect VLP sequences in milk samples, as one would assume that populations would be in very low numbers in these substrates. This point seems especially relevant given >25% of the sequences identified in the faecal viral assemblage were most similar to those of phages infecting lactic acid bacteria known to be abundant in breast milk. It is not known from where the initial infant gut virome originates, although it has been suggested dietary, environmental and maternal sources may be sources of inocula.^32^ In addition, primary infant faecal viruses may originate from induction of prophages from primary bacterial colonisers.^32^

A more‐extensive metagenomic study examining the infant (DNA and RNA) virome in four pairs of twins over the first 24 months of life (sampling at 0, 3, 6, 9, 12, 18 and 24 months) identified eukaryotic DNA viruses from month 3 onwards (ssDNA—anelloviruses, ciroviruses, geminiviruses, nanoviruses, parvoviruses; dsDNA—adenoviruses, polyomaviruses), but only anelloviruses, circoviruses and geminiviruses were detected in at least one twin of each pair between months 3 and 24 of the study.^46^ Anelloviruses, associated with host immune status, were most prevalent from 3 months of age and highly divergent from known anelloviruses, with their abundance peaking at 6‐12 months of age. The same anelloviruses were detected in faecal samples of the same infant collected 12 months apart, suggesting a persistent or stable source of recurring infection.^46^ It was speculated the increase in anelloviruses between 6 and 12 months was the result of lowered immune status associated with the decrease in maternal IgG, though no data were provided to support this. Eukaryotic viral (DNA) richness was low at 3 months but increased thereafter, indicating environmental exposure contributes to the establishment of this component of the virome. Eukaryotic RNA viruses (ssRNA—alphaflexiviruses, astrovirsues, caliciviruses, picornoviruses, tombamoviruses, virgaviruses; dsRNA—chrysoviruses, picobirnaviruses) were detected less frequently across the study period, but related twins harboured the same strains of parechovirus and enterovirus. Phages were detected at high abundance in all infants across the study period, with the majority of reads belonging to the order *Caudovirales* (*Siphoviridae* > *Myoviridae* > *Podoviridae*), *Inoviridae* (ssDNA), *Microviridae* (ssDNA), *Corticoviridae* and *Tectiviridae*. No RNA phages were detected in any of the faecal samples. Phage richness and diversity were greatest at month 0 and decreased as the infants aged, and the relative abundance of *Caudovirales* (high at 0 months, low at 24 months) was inversely correlated with the abundance of *Microviridae*. crAssphage^22^ was detected in one sample of one infant, suggesting this phage is not acquired early in life. Of note, reads associated with the family *Lipothrixviridae*, an archaeal virus family, were detected in the faecal virome of all twins at various points across the study period. Each infant had a unique faecal virome, though the viromes of related infants were more similar to one another than nonrelated infants after controlling for age. Bacterial species richness and diversity increased with age, being predominated by *Firmicutes*,* Bacteroidetes*,* Actinobacteria*,* Verrucomicrobia*,* Proteobacteria* and *Fusobacteria* at 24 months. An inverse correlation of phage and bacterial richness (high phage–low bacteria to low phage–high bacteria) was seen from month 0 to 24 months. The infant faecal virome is suggested, therefore, to display a reversed predator‐prey cycle: ie, limited prey diversity controls predator abundance.^46^ The initially high phage population is unsustainable because of low numbers of bacteria colonising the GI tract. Consequently, the phage assemblage shrinks in size and diversity, relieving pressure on the bacterial community and allowing it to establish and colonise the gut.

## 12 INFLUENCE OF ENVIRONMENTAL FACTORS ON DEVELOPMENT OF THE INFANT VIROME

Differences in birth mode (Caesarean vs vaginal delivery) and diet (breast‐ vs formula‐feeding; age at which weaning began) were confounders in the study of Lim et al.^46^ Differences in the faecal microbiota of breast‐ and formula‐fed infants are well documented, as are the effects of weaning.^47^ It is also known that 16S rRNA gene‐based sequencing studies do not always give accurate representation of *Bifidobacterium* populations in the infant faecal microbiota.^48^, ^49^ Therefore, shotgun metagenome studies examining the infant microbiome and virome are required in carefully controlled populations to study the acquisition and development of the infant GI virome.

The influence of malnutrition on development of the infant faecal virome was investigated during the first 30 months of life in eight pairs of healthy twins and 12 pairs of twins discordant for severe acute malnutrition (six pairs with one twin healthy, one with marasmus; six pairs with one twin healthy, one with kwashiorkor).^50^ As with the study of Lim et al^46^ *Anelloviridae* were prevalent in early life, but diminished at 15‐18 months of age. In addition, *Microviridae* increased in abundance with age. *Siphoviridae* were the most abundant VLPs during 0‐10 months, but then slowly decreased. This demonstrated that, as with the prokaryotic component of the faecal microbiota, age affects virome composition in early life. Discordant twins could be distinguished from healthy twins based on phage populations and the presence of higher levels of eukaryotic viruses of the families *Anelloviridae* and *Circoviridae*.

## 13 THE INTESTINAL VIROME, IMMUNE RESPONSE, AND DISEASE

Alterations in the intestinal virome characterised by reduced diversity and altered intestinal barrier function have been associated with infectious and autoimmune diseases in addition to metabolic disorders and cardiovascular disease (Figure 1). A potential pathogenic mechanism involves translocation of viruses present in the gut microbiota across the intestinal epithelial barrier, particularly in situations of altered and increased permeability that exert immunomodulatory effects on the host.^51^

HIV‐1 and rotavirus are known to escape the gut and disseminate to peripheral tissues via components of the immune system.^51^ Enterocytes are able to recognise specific phage peptides and transport them across the mucosal barrier.^51^ HIV patients are affected by translocation, and have associated systemic inflammation.^52^ Changes to the faecal virome and bacterial microbiome have been seen in HIV patients, with increased numbers of adenovirus‐associated sequences and increased bacterial diversity and richness associated with peripheral CD4 T‐cell counts of <200; phage populations are not detectably affected by HIV disease status or anti‐retroviral therapies,^52^ but plasma samples from HIV patients contain gut microbiota‐derived nucleic acids.^53^ Phages have been detected in blood samples of healthy humans, while mycobacteriophages have been found in the blood of patients with sarcoidosis, and Crohn's disease (CD), and in healthy individuals.^51^, ^54^ Circulating phages have also been detected in blood samples after oral administration of antibiotics to patients with bacterial infections, whereas no phages could be detected in their blood prior to antibiotic therapy.^51^ It is not known if these phages were of GI origin. The presence of phages in the peripheral blood has been termed “phagemia”.^51^ However, whether the blood contains its own “baseline” VLP population to which the GI virome can contribute in disease is unknown.

Human‐associated viruses and intact phages and/or their nucleic acids are immunogenic. Newborns and children vaccinated with phage (ϕX147) formed antibodies against the phage within 1 week of exposure.^55^ Tumour‐specific phages can be used to treat disease by initiating infiltration of neutrophilic granulocytes in a toll‐like‐receptor (TLR)‐dependent manner.^56^ Mono‐association of germ‐free mice with *Murine norovirus* (positive strand RNA virus) was sufficient to replicate the beneficial effects of commensal bacteria, restoring small‐intestinal morphology and lymphocyte function without overt inflammation or disease.^57^ Presence of innate lymphoid cells was reduced in the virus‐treated mice compared with controls. Inoculation of antibiotic‐treated, wild‐type mice with *Murine norovirus* for 10 days increased villi width, Paneth cell granules, intestinal T cells and IFN‐γ expression, producing comparable but nonidentical results to when antibiotic‐treated mice were inoculated with strains of commensal bacteria. Intestinal gene expression was affected by *Murine norovirus* inoculation, influencing lymphoid cell development and immune responses mostly related to the anti‐viral type I interferon (IFN‐I) response; IFN‐I signalling was necessary for virus‐mediated improvements in the *Murine norovirus*‐infected mice. Genes associated with development and function of haematopoietic cells were also affected. Inoculation of antibiotic‐treated mice with *Murine norovirus* protected the mice from *Citrobacter rodentium* super‐infection. The protective effects of the virome may explain recent findings with respect to treatment of patients with *Clostridium difficile* infections with sterile faecal filtrates: based on the published protocol, the faecal filtrates used in the study are likely to have contained phages in addition to the “bacterial debris, proteins, antimicrobial compounds, metabolic products, and oligonucleotides/DNA” reported.^58^ Support for a role for phages in the success of faecal microbial transplants (FMTs) to treat *C. difficile* infections comes from a pilot study by Zuo et al^59^: when donors had higher diversity (richness) of *Caudovirales* than recipients, FMT was successful; in instances where recipient virome richness was greater than that of the donor, over half of the recipients experienced disease recurrence after FMT. Large cohort studies that characterise donor and recipient viromes are required to validate these findings.^59^

## 14 THE INTESTINAL VIROME AND INFLAMMATORY BOWEL DISEASE

The most compelling and direct evidence for enteric viruses playing a causal role in chronic GI inflammation, however, comes from studies in mice.^60^ Mice with a mutation in the autophagy gene *Atg16L1*, which in humans predisposes to CD, develop normally and are symptom‐free. However, enteric infection by norovirus resulted in the manifestation of the disease, although solely having the virus or the susceptible allele alone did not produce the disease, demonstrating the importance of virus‐susceptibility gene interactions in disease progression. Using a dextran sulphate sodium (DSS)‐induced rodent model of colitis, importance of the gut virome to GI immune homeostasis was demonstrated.^61^ In mice administered a cocktail of anti‐viral drugs followed by DSS, the animals treated with anti‐virals had much more severe colitis than the animals with an intact virome, along with greater weight loss and reduction in colon length. Protective effects of the virome were mediated by stimulation of both TLR3 and TLR7, but not individually. Administration of inactivated rotavirus partially restored the virome‐associated protective effect to the anti‐viral‐treated mice. TLR3/TLR7‐deficient mice were more sensitive to colitis than their wild‐type counterparts. A link to human inflammatory bowel disease (IBD) is the observation that patients carrying mutations in both TLR3 and TLR7 had higher rates of hospitalisation compared with IBD patients without mutations.^61^

## 15 THE INTESTINAL VIROME IN HUMAN IBD

A link between the gut virome and CD was first suggested when it was found CD patients (n = 19) had significantly more VLPs (phages) in colonic biopsies than healthy individuals (n = 10) (2.9 × 10^9^ vs 1.2 × 10^8^ VLPs/biopsy), and for the CD patients ulcerated mucosa had significantly fewer VLPs than nonulcerated mucosa (2.1 × 10^9^ vs 4.1 × 10^9^ VLPs/biopsy).^9^ More recent, small‐scale studies have been undertaken to characterise the gut virome of IBD.^29^, ^62^, ^63^, ^64^ A pilot metagenomic study of ileal and colonic contents of six CD patients and ileal samples from six non‐IBD controls showed CD DNA‐based viromes had higher phage abundance than control samples.^62^ However, the ways in which virome data from healthy controls (n = 8) and patients with ileocolic CD (n = 11) were analysed were found to contribute to interpretation of results.^63^ Prophages represented the most hits in the CD samples when unassembled reads were compared, but were higher in the control group when the assembled data were compared. Analysis based on assembled data showed fewer differences between the CD and control group. Nonrarefied data were used to generate estimators of species richness: the viromes of CD patients were less diverse than the healthy patients, but showed greater heterogeneity across samples. The CD and control samples could not be fully separated into two groups based on VLP composition and abundance.

A later DNA‐based study showed differences in CD patients related to disease status (newly diagnosed, active onset, active pre‐surgery) and therapy.^65^ Similar to their previous study, individual variability and sample origin had a greater effect on the virome than CD, although more over‐represented viruses were found in the CD viromes than the healthy, and newly diagnosed patients had higher diversity in faecal and biopsy viromes than those with active disease. Patients on steroids and/or immunosuppressors had lower diversity than untreated patients, while those on immunosuppressors only had lower diversity than those on combination therapy or steroids only. The study benefitted from recruiting CD and ulcerative colitis (UC) patients and their family members in the UK (Cambridge) and USA (Los Angeles), thereby controlling for household factors that may influence the microbiome.^29^ An increase in phage‐associated richness (predominantly *Caudovirales* and *Microviridae*) was seen in IBD faecal viromes compared with those of controls. Initial findings were confirmed using two independent and geographically distinct US (Boston, Chicago) patient cohorts with matched controls.^29^ It was suggested decreases in bacterial richness concomitant with increases in phage richness may be due to predator‐prey dynamics, but it was unclear how these contribute to the pathogenesis of IBD. Also proposed was the virome as a target for therapeutic modulation.^29^ Although sharing some VLPs, CD and UC viromes could be separated from one another and harboured unique phages. Phages of *Lactococcus*,* Lactobacillus*,* Clostridium*,* Enterococcus* and *Streptococcus* were significantly associated with disease. Similar to other studies,^27^, ^38^ there was also conservation of VLPs among members of the same household. Manrique et al^28^ found the HGP was perturbed in the UC (*n* = 66) and CD (*n* = 36) viromes of Norman et al^29^: healthy subjects harboured on average 62% of the 23 core phages, while this number was significantly reduced (37% and 30%, respectively) in UC and CD patients.

Examination of RNA viruses present in colonic tissue from 10 IBD (6 CD, 4 UC) and five healthy individuals demonstrated IBD patients carried more mammalian viruses and human pathogens than healthy individuals; *Adenoviridiae* were present in the IBD samples but absent from the healthy controls.^64^ HERV (human endogenous retroviruses) were found in the IBD viromes; expression of HERV proteins has been linked with inflammatory conditions. HERV was present at significantly higher levels in IBD patients co‐infected with herpesviruses. The significance of this association with respect to IBD is unknown, but infection with other viruses may trigger expression of HERV.^64^

## 16 CHALLENGES FACING STUDIES OF THE HUMAN INTESTINAL VIROME

Metagenomic studies of the virome are not without their limitations or challenges. Many of these are related to isolation and processing methods. The majority of studies published to date rely on the use of 0.2 μm filters, but it is known these halve the number of viruses detected compared with use of 0.45 μm filters and will not detect giant viruses.^10^, ^66^ Consequently, we do not have a true picture of the diversity of the human virome.

### 16.1 Sequencing depth

Characterisation of total faecal metagenomes has, to date, relied upon generating ~5 Gb of sequence data per sample to allow full characterisation of microbiomes.^15^, ^16^, ^17^, ^18^ With the exception of a recent study that generated 5‐8 Gb sequence data per sample,^59^ the most extensive surveys of virome diversity have sequenced only a fifth of this depth per sample, with many relying on much lower sequencing depths for characterisation of viromes. With the exception of the four viromes generated by Manrique et al^28^ all viromes generated to date have relied upon PCR amplification or multiple displacement amplification (MDA) to generate sufficient DNA for sequencing. These methods introduce bias into sequencing libraries. For example, when using the WTA2 kit it is recommended to use the Nextera XT library preparation kit to avoid cluster‐calling problems during Illumina sequencing. However, the Nextera kit causes under‐representation of ends of viral genomes because of the way this kit works.^66^ CsCl centrifugation has been shown to be the most efficient method for removing host DNA, but it discriminates against some phages and is not reproducible; consequently, this method is not suitable for quantitative studies.^67^

### 16.2 Phage isolation

Methods of recovery of faecal phages have improved recently (Table 2), so it should be feasible to generate PCR‐ or MDA‐free, deep‐sequenced viromes to allow greater characterisation of the human gut virome. The question will then become how many sequence data are required to adequately characterise the virome. Current sequencing depths and paucity of available virus genome sequences (Figure 2) may mean phages that infect different bacterial hosts may be indistinguishable.^20^ Low sequencing depth also means low coverage of VLP genomes within samples, making it difficult to differentiate between spurious alignments and real virus sequences and potentially leading to overestimation of VLPs in samples when relying on automated read alignments.^68^ Bioinformatics tools are required that allow differentiation of authentic sequences from artefacts.

### 16.3 Isolation‐kit contaminants

Few studies of the human virome have considered the effects of contaminants in reagents and nucleic acid extraction kits on viral metagenomes (Table S1), though these are being increasingly recognised in prokaryote‐focused studies.^26^, ^69^, ^70^ Nucleases, proteases and polymerases used in studies are produced in protein expression systems, and sequences associated with these expression vectors have been detected in virome studies.^70^ Columns used to purify DNA may introduce parvovirus‐like, circoviruses/densoviruses and iridoviruses sequences into samples, and it has been suggested their use should be avoided in virome studies.^26^, ^70^ Efforts should be made to prevent cross‐contamination of samples when processing samples for nucleic acid extraction and sequencing.^26^, ^70^ Low‐biomass samples obtained from non‐GI sites will be reliant on PCR‐based approaches for the foreseeable future, and these are most likely to be affected by contaminants in reagents. Therefore, appropriate negative controls should be included in sequencing studies, to allow the identification of sample‐associated sequences rather than those from contaminants.

### 16.4 Combining virus isolation and metagenomics

Those studies targeting only the VLPs of the microbiota will not capture prophage diversity within samples, and will contain little information to allow studies of phage‐host interactions or host ranges, limiting in‐depth analyses of the overall ecology of the gut microbiome.^21^ Studying total community metagenomes in conjunction with VLP‐derived metagenomes will provide a more‐complete picture of virus‐host interactions in the human gut.^21^ The greatest challenge to integrated studies of the whole gut microbiome is the lack of available phage sequences from isolated viruses (Figure 2). Because most nucleotide sequences obtained from viral metagenomes are novel, BLASTN analyses are generally uninformative.^71^ It has been stated that isolating “just a few phage genomes from novel environments will greatly increase our understanding of viral diversity in these environments”.^72^ Recently, OligoNucleotide Frequency profiling, in conjunction with abundance profiling and homology methods, has been proposed as a means of improving identification of microbial hosts for novel viruses from total community metagenomes^73^, ^74^; deriving full benefit from this approach relies on classical isolation and genomic characterisation of viruses and their hosts from environmental samples. Therefore, it is of utmost importance that isolation of phages from the GI tract and their genomic characterisation are at the forefront of future research: incorporation of ecological studies into those exploiting lytic phages or phage‐encoded gene products for biotechnological or medical applications would be of great benefit to the research community.

### 16.5 Environmental confounders

As with the prokaryote component of the microbiome, the genetic makeup of an individual's virome is influenced by diet, nutrition status, health, socioeconomic group, geographical location, age, lifestyle, season and medication.^7^, ^20^, ^29^, ^50^, ^75^ Immune status, and viral and human genetics will also affect interactions of the virome with the host.^29^, ^52^, ^75^ Virome studies have so far focused mainly on small human cohorts from restricted geographical locations, and to a large extent have focussed on characterising phage populations. As of July 2017, there are ~2040 complete genome sequences available for the *Caudovirales*, the main order of phages (Figure 2). Our coverage of eukaryote‐associated infectious viruses is equally poor, mostly because we do not (1) know how to propagate known infectious agents and (2) there are a number of medical conditions speculated to be linked with viral infections (eg, type I diabetes, chronic fatigue syndrome, obesity) but for which no infectious agent has been found. It has been estimated over half of human‐infecting viruses remain to be discovered.^76^ Numerous conditions in which patients suffer unexplained symptoms may also be virus‐associated. Therefore, there is a need to sample a wide range of body fluids and tissues from individuals globally to better understand the role of viruses in human health and disease.^75^ Propagation and characterisation of prokaryote‐ and eukaryote‐infecting viruses will require huge efforts to fully characterise extant viral populations, and there is still a wealth of viral diversity (pathogenic and nonpathogenic) to be discovered within human viromes. Those in areas with poor sanitation and crowded living conditions, travellers, children and the immunocompromised are expected to have higher viral loads, at least in terms of potentially pathogenic viruses,^75^ while pre‐term infants, breast‐ and formula‐fed infants and the elderly are likely to have very different viromes compared with adults based on the general information we have about the composition of the human gut microbiome throughout life.^27^, ^50^ Interactions with pets, insects and wild animals will also influence the composition of the human virome. From a pathogen perspective, bush‐workers, abattoir workers and individuals exposed to insect bites have been highlighted as likely sources of new human‐infecting viruses.^75^

With the advent of high‐throughput, inexpensive, sensitive sequencing methods, it has become possible to study the contribution of dsDNA, ssDNA and RNA VLPs to the human virome. Gene transfer agents, which appear to represent defective phages, have not been examined in the context of the human microbiome or virome, but may also make a contribution to its genetic content.^77^, ^78^ Similarly, exosomes—extracellular vesicles of 40‐100 nm in diameter consisting of proteins, lipids, miRNA, mRNA and DNA—derived from cells lining the GI epithelium are likely to contribute to nucleic acids found in viromes.^79^, ^80^ Outer‐membrane vesicles, the equivalent (in size and composition) of eukaryotic exosomes for Gram‐negative bacteria, may also contribute to what we currently call the virome, as these vesicles have recently been reported to contain DNA as well as RNA.^81^

### 16.6 Virome‐specific bioinformatics tools

Whereas 16S rRNA gene sequence data are readily processed and analysed using packages such as QIIME and Mothur, no such tools exist for the analyses of sequence data derived from host‐associated viromes.^35^ Tools exist for functional annotation of viromes and estimating viral diversity (Table 3). No easy‐to‐use pipeline that takes raw reads, strips out host DNA, looks for bacterial contaminants then assigns taxonomy and functionality to prokaryote and eukaryote viruses within samples exists, though efforts are being made to generate such tools. These will need to take into account the presence of human endogenous retroviruses, which comprise ~8% of the human genome and still possess the ability to encode retrovirus polymerase and envelope protein and can be reactivated by exogenous retroviruses such as HIV.^53^

**Table 3:** Available tools for analysis of viruses in total community and viral metagenomes

Name	Description	Website/reference(s)
PHACCS^89^	Assesses the biodiversity of viromes; requires knowledge of virus genome sizes; desktop app	https://sourceforge.net/projects/phaccs/
ACLAME^90^	Classification of mobile genetic elements; web‐based	http://aclame.ulb.ac.be
MetaVir 2^91^, ^92^	Annotates viral metagenomes (raw reads or assembled contigs); web‐based	http://metavir-meb.univ-bpclermont.fr
VIROME^93^	Classification of predicted open‐reading frames (ORFs) from viral metagenomes; each ORF placed into one of 9 sequence categories; functional classification; web‐based	http://virome.dbi.udel.edu
Vanator^94^	Perl pipeline using a number of alignment, assembly and analysis tools to assess metagenomic data derived from Illumina data; virus discovery; desktop app	https://sourceforge.net/projects/vanator-cvr/
VirSorter^95^	Detects virus signals in single‐cell amplified genomes of uncultivated organisms or genomic fragments assembled from metagenomic sequencing; desktop app	https://github.com/simroux/VirSorter
ViromeScan^96^	Characterises taxonomy of eukaryotic viruses directly from raw reads; desktop app	https://sourceforge.net/projects/viromescan/
VIP^97^	Identification of eukaryotic viruses (pathogens, influenza virus) from metagenomes; desktop app	https://github.com/keylabivdc/VIP
VirusDetect^98^	Analyses small RNA datasets for known and novel viruses; web‐based and desktop app	http://bioinfo.bti.cornell.edu/tool/VirusDetect/
PHASTER^99^	PHAge Search Tool—Enhanced Release, rapid identification and annotation of prophage sequences within bacterial genomes, plasmids and metagenomic sequences	http://phaster.ca/
VirusSeeker^100^	BLAST‐based sequence analysis pipeline for eukaryotic and prokaryotic virus discovery and virome composition; desktop app	https://wupathlabs.wustl.edu/virusseeker/
VirHostMatcher^74^	Computes Oligonucleotide Frequency (ONF) scores between viral and host sequences in total community metagenomes, and visualises results; higher predictive accuracy at class and phylum levels	https://github.com/jessieren/VirHostMatcher
VirFinder^73^	k‐mer frequency‐based, machine‐learning method for virus contig identification in total community metagenomes	https://github.com/jessieren/VirFinder

## AUTHORSHIP

*Guarantor of the article*: Simon R. Carding.

*Author contributions*: SRC takes responsibility for the integrity of the work as a whole, from inception to published article. LH and SRC designed the research study. LH and ND collected and analysed data. SRC and LH wrote the paper. All authors approve the final version of the manuscript.
