# Supplementary material for: Review article: the human intestinal virome in health and disease
Source: Aliment Pharmacol Ther. 2017 Sep 4;46(9):800–15. doi: 10.1111/apt.14280 (PMC5656937; doi:10.1111/apt.14280)
Supplement: Supplementary file 1 [file APT-46-800-s001.docx]

**Supporting information**

**Table 1.** Virome studies conducted on non-intestinal-associated human viromes

| **Body site(s) sampled** | **Main findings** |
| --- | --- |
| Blood (plasma) from healthy donors^54^ | Rapid method for recovering viruses from blood samples  Recovered sequences from viruses known to infect humans, and dsDNA and ssDNA viruses  Recovered sequences with significant similarities to four different bacteriophages (ϕ3626, EJ-1 psiM2 and ϕCPAR39)  Adaptation of method may allow identification of RNA viruses in samples |
| Sputum samples from cystic fibrosis (CF) and non-CF individuals, and an asthma sufferer^37^ | Core set of 19 phage genomes thought to be characteristic of the human respiratory tract identified  12 genomes present in the CF viromes were absent from the non-CF viromes  Metabolic profile of the CF-associated virome harboured specific adaptations that facilitated survival in the CF respiratory tract  Species richness of the CF virome lower than that of the non-CF virome  Spouse of a CF individual had a CF-like virome: first demonstration of exchange of phages between individuals via a shared external environment  Asthma-associated virome similar to the CF virome; due to the highly similar respiratory pathologies of the two conditions (impaired mucociliary clearance, extended periods of persistence of bacteria in disease states compared with healthy) |
| Sputum of cystic fibrosis patients and unaffected individuals^104^ | Using data from Willner *et al.*^37^, found significant enrichment of sequences associated with efflux pumps and antimicrobial resistance (fluoroquinolone, β-lactamase) in CF patients compared with controls |
| 19 pooled oropharyngeal samples^105^ | Virome comprised phages, Epstein–Barr Virus only eukaryotic virus detected  Recovered complete genomes of *Escherichia coli* phage T3, *Propionibacterium acnes* phage PA6 and *Streptococcus mitis* phage SM1  Demonstrated induction of *S. mitis* SF100-encoded SM1 by nicotine and soy sauce  Phage-encoded platelet-binding factors of SM1 (*pblA*, *pblB*) found in oropharyngeal and salivary viromes |
| Saliva of healthy individuals^106^ | Identified temporally distinct phage populations in saliva (60- to 90-day period)  1×10^8^ VLPs/mL saliva  Shared phage populations in individuals living together  Salivary viruses may be a reservoir of virulence factors with pathogenic functions in the oral cavity  Demonstrated distinct salivary, faecal and respiratory viromes |
| Plasma from Chinese HIV/AIDS patients and uninfected individuals^53^ | Bacteria detected in blood similar to gut microbiota, suggesting acquisition of microbial elements (bacteria and/or nucleic acids) from the human gut  HIV/AIDS patients had phage (predominantly enterobacteria and *Pseudomonas*) sequences in their blood along with anelloviruses  Only anelloviruses were found in the plasma of healthy individuals |
| Plasma samples from HIV-infected patients in US and Uganda^107^ | Compared plasma DNA and RNA viromes of patients with low and high CD4^+^ T cell counts  Detected HIV, hepatitis C virus, hepatitis B virus, GB virus C, anellovirus, and human endogenous retrovirus in samples  Increased number of reads associated with anelloviruses in US patients with low CD4^+^ T cell counts, suggesting AIDS associated with reduced control of anellovirus replication and higher viral loads  Detected contaminants associated with the reverse transcriptase used to generate cDNA, and sequences related to small circular and linear single-stranded DNA (ssDNA) genomes and iridoviruses that have been reported to contaminate nucleic acid columns used in nucleic acid extractions |
| Plasma of heart and lung transplant patients on immunosuppressants and anti-virals^108^ | Longitudinal study of 96 transplant patients (656 samples collected in total)  Included sequencing controls in their study to rule out contaminants contributing to findings  Virome affected by post-transplant therapies, but bacteriome not; viruses more abundant than bacteria  Virome composition converged to drug-determined state  Increase in presence of anelloviruses (Torque Teno viruses) upon immune suppression  Anellovirus burden lower in rejecting transplant recipients than in non-rejecting |
| Plasma from patients with chronic hepatitis B, chronic hepatitis C, autoimmune hepatitis (AIH), non-alcoholic steatohepatitis (NASH) and controls^109^ | RNA and DNA viruses studied (7 samples each in total)  Detected expected viruses in patients, but also additional ones  Low number of patients used limit usefulness of study for associated viruses found with AIH or NASH  Novel circovirus identified, presence confirmed by PCR, cloning and sequencing  Proposed methods used in study could be extended to other bodily fluids |
| Saliva^110^ | Longitudinal sampling (8 individuals, 11 time points across 60 days)  Phages predominated in the viromes  Sex-specific differences in viromes, with persistence of some (~60 %) viruses over two or more time points |
| Nose, skin, mouth, vaginal and faecal samples^111^ | Examined metagenomic datasets from the Human Microbiome Project for eukaryotic viruses  Each individual had a distinct viral profile: some viruses stable in virome, others transient  15 viral genera detected (average of 5.5 viral genera in each individual)  dsDNA viruses belonged to the families *Herpesviridae*, *Papillomaviridae*, *Polyomaviridae*, and *Adenoviridae*  ssDNA viruses belonged to the families *Anelloviridae*, *Parvoviridae*, and *Circoviridae*  Numerous undescribed human papillomaviruses may exist  Anelloviruses found in all body sites except faeces  Most components of oral virome shared with faecal virome |
| Allograft bronchoalveolar lavages and oropharyngeal washes of lung transplant, healthy and HIV-positive subjects^112^ | DNA virome  Significantly more anelloviruses in BAL of transplant patients than in health and HIV-positive subjects  0.75 % of total reads for all samples combined assigned to bacterial phages  81 % of reads had no database matches  Findings validated using qPCR with primers designed from assembled contigs  High levels of anelloviruses correlated with dysbiosis of the bacterial component of the lung microbiota |
| Nasopharyngeal aspirates and sputum of children with respiratory infections and controls^70^ | Detection of DNA and RNA viruses in samples (human rhinovirus C, entero- and rhinoviruses, human metapneumovirus, human respiratory syncytial virus, anelloviruses)  Detected phage sequences  Almost-complete viral genomes assembled with reads from infection-positive samples  Demonstrated heterogeneities in human rhinovirus type C sequence limit usefulness of PCR compared with metagenomics for detection of infectious agent  Highlighted issue of contaminants in virome studies |
| Skin samples from 8 body sites of 16 subjects^113^ | Two samples taken, one month apart, for each site/individual; skin virome not stable over time (<50 % shared over time)  Included negative and mock communities as sequencing controls  Phages (*Caudovirales*, >85 % temperate) predominated (variable *Propionibacterium*, *Staphylococcus*, *Pseudomonas*, *Bacillus*), with papillomavirus found on palm samples  ~0.4 % of skin total metagenome reads associated with viruses  Natural skin occlusion strongly associated with skin virome diversity |
| Serum of 569 individuals across four continents^114^ | VirScan, high-throughput method for analysing antiviral antibodies  Phage-immunoprecipitation sequencing (PhIP-seq) of over 10^8^ antibody–peptide interactions involving proteins of 206 different viruses and over 1000 different strains  Detected average of 10 viruses in each person  Could potentially be applied to detect immune responses to virome-associated phages in future |
| Urine from 10 people with urinary tract infections (UTIs), 10 without^115^ | Detected ~10^7^ VLPs/mL urine  Phages most predominant viruses, but eukaryotic viruses (papillomavirus widespread) also present  No association between UTIs and virome diversity, even though bacterial communities differed |
| Blood from patients pre- and post-transfusion, and haemophiliacs treated with clotting factor^116^ | Characterized a novel human virus, *Human hepegivirus 1* (HHpgV-1), present in serum from two blood transfusion recipients and two haemophilia patients who had received plasma-derived clotting factor concentrates |
| Blood of donors with elevated serum alanine aminotransferase (ALT) levels^117^ | Analysed blood from patients with 79 U/L (*n* = 100), 61–79 U/L (*n* = 100) and <61 U/L (*n* = 100) ALT  No statistically significant difference among groups based on viruses (*Herpesviridae*, *Anelloviridae*, *Picornaviridae*, and *Flaviviridae*) detected in samples; therefore, serum ALT levels not suitable indicator for potential transfusion-transmitted infections |
| Plasma of 498 Kenyan adults in 51 pooled samples^118,119^ | 14 different viruses [parvovirus B19, pegivirus C (GBV-C), alpha anellovirus, gamma anellovirus, DENV-2, beta anellovirus, HIV-1, HBV, rhinovirus C, Kadipiro virus (KDV), MCPyV ,HHV-6, rotavirus, norovirus] detected in pooled samples  Highlighted contaminants in two different RNA extraction kits  Corrigendum: KDV RNA detected may be a sporadic contaminant of one of the reagents or of the QIAamp Viral RNA Mini Kit used |
| Bronchoalveolar lavage fluid and blood samples from lung-transplant recipients, donors and healthy controls^68^ | Higher and lower levels of Torque teno viruses in respiratory tract and blood, respectively, of lung-transplant recipients and donors compared with controls  Donor lungs abnormal with respect to virome populations, even before transplantation |
| Conjunctiva of healthy individuals^120^ | There is a resident DNA viral community on the ocular surface, with Torque teno virus predominating |
| Neonatal blood spots of children who developed acute lymphoblastic leukaemia and unaffected controls^121^ | Detection of human herpesvirus type 6, human endogenous retroviruses and *Propionibacterium* phage in low numbers of patients; first two viruses likely to be contaminants from integration into human DNA, the *Propionibacterium* phage may be from skin contaminants  DNA viruses did not have a major role in development of acute lymphoblastic leukaemia |
| Plasma from individuals at high risk of parenterally and sexually transmitted infections^26^ | Anelloviruses (Torque teno virus, Torque teno mini virus, Torque teno midi virus/small anellovirus) found in all pooled samples  Human pegivirus detected in high number in HIV-infected subjects  Highlighted issues of contaminants, cross-contamination during library preparation and inability to detect low viral loads in virome studies |
| Blood of 8,240 individuals^122^ | Mapped sequences to 94 different viruses, with 75 of these representing contaminants associate with reagents and the environment  19 human DNA, proviruses and RNA viruses detected in 42 % of individuals  Identified Merkel cell polyomavirus in 49 individuals, papillomavirus in blood of 13 individuals, parvovirus B19 in 6 individuals, and herpesvirus 8 in 3 individuals; age, sex and ancestry contributed to prevalence of infection |
